# DNA methylation profiles aid to identify putative genome activation histories along lymphomagenesis

**DOI:** 10.1093/nargab/lqaf187

**Published:** 2026-01-10

**Authors:** Leone Albinati, Irene D’Onofrio, Rabia Gül Aydin, Houyem Toukabri, Renée Beekman

**Affiliations:** Department of Genome Biology, Centre for Genomic Regulation (CRG), The Barcelona Institute of Science and Technology (BIST), Barcelona 08003, Spain; Department of Medicine and Life Sciences, Universitat Pompeu Fabra (UPF), Barcelona 08003, Spain; Department of Genome Biology, Centre for Genomic Regulation (CRG), The Barcelona Institute of Science and Technology (BIST), Barcelona 08003, Spain; Department of Medicine and Life Sciences, Universitat Pompeu Fabra (UPF), Barcelona 08003, Spain; Department of Pharmacy and Biotechnology, University of Bologna, 40126 Bologna, Italy; Department of Genome Biology, Centre for Genomic Regulation (CRG), The Barcelona Institute of Science and Technology (BIST), Barcelona 08003, Spain; Department of Medicine and Life Sciences, Universitat Pompeu Fabra (UPF), Barcelona 08003, Spain; Department of Genome Biology, Centre for Genomic Regulation (CRG), The Barcelona Institute of Science and Technology (BIST), Barcelona 08003, Spain; Department of Genome Biology, Centre for Genomic Regulation (CRG), The Barcelona Institute of Science and Technology (BIST), Barcelona 08003, Spain; Department of Medicine and Life Sciences, Universitat Pompeu Fabra (UPF), Barcelona 08003, Spain; Lymphoid Neoplasms Program, Institut d’Investigacions Biomèdiques August Pi i Sunyer (IDIBAPS), Barcelona 08036, Spain

## Abstract

DNA methylation (DNAm) is widely used to leverage biological information in the context of cancer. Active regulatory elements marked by increased chromatin accessibility and specific transcription factor (TF) binding, such as enhancers, show tumor-specific DNAm patterns reflecting the biological forces shaping tumorigenesis. However, DNAm changes also occur at regions that are inactive at histone-mark level in fully developed tumors. Beyond hypermethylation of promoters, these changes are often overlooked, leaving their functional relevance poorly understood. By analyzing DNAm and chromatin state annotations from conventional mantle cell lymphoma (cMCL), we identified a subset of ~300 CpGs with homogeneous cMCL-specific demethylation patterns located in cMCL-inactive regions. We show that these regions contain cMCL-related TF motifs and are flanked by genes with cMCL-specific expression patterns, suggesting a potential regulatory role in lymphomagenesis. We hypothesize that these regions represent DNA demethylation imprints of genome activation prior to full-blown tumor formation, either present in its cell type of origin (COO) or during early stages of tumor formation. Altogether, we put forward a new, DNAm-centered approach to gain insights into potential early tumorigenic events, allowing us to generate novel, further explorable hypotheses to better understand the features playing a role in lymphomagenesis and beyond.

## Introduction

DNA methylation (DNAm) landscapes harbor a vast amount of information that has started to become unraveled in the past decades. First, it is now commonly accepted that by focusing on cell-type-specific enhancer regions, DNAm loss can be leveraged to uncover cellular identities in the context of normal differentiation and malignant transformation [1–5]. Second, analyses centered around CpG islands at promoter regions enable us to identify tumor suppressor genes in cancer samples [6]. And third, DNAm at heterochromatic and polycomb-associated regions harbors information to read out proliferative histories [7]. Altogether, this shows that dependent on the (epi)genomic context, e.g. in enhancers, promoters, repressed loci, or heterochromatic regions, CpG DNAm levels can provide different types of biological information. Previous studies have furthermore shown that DNAm does not only harbor information regarding enhancer activation at present but can also inform about past activity states. DNAm loss at regulatory elements has specifically been shown to be mainly unidirectional during differentiation and persists even after chromatin activity is lost [8]. Moreover, in the context of development, each cell type harbors a DNA demethylation imprint of its germ layer-specific enhancers. These enhancers are no longer active in these cells, but the germ layer-specific DNA demethylation signals at these regions remain [9]. Another example is pregnancy-driven loss of DNAm at regulatory elements in mammary glands, which persists after pregnancy when the regulatory elements are decommissioned [10]. This feature makes DNAm patterns clearly distinct from transcriptomic information that reflects current but not past cellular identities. In other words, DNAm data harbor a powerful extra dimension that can be used to understand cellular histories. Overall, the complex (epi)genome-context-related and spatiotemporal aspects outlined above make DNAm analyses challenging and promising at the same time, leaving a wealth of knowledge still to be uncovered.

A particular area in which DNAm data can help to better understand biological processes is the study of tumorigenesis. More specifically, this epigenetic layer can aid in discovering tumor suppressor genes, identifying tumor-specific enhancer regions, reading out cellular proliferative histories, and tracing the tumoral cell type of origin (COO) [11]. The latter is particularly relevant to better understand from which healthy cell subtype a tumor originates. In this study, we explored how DNAm could be leveraged to identify putative cellular processes that are active during early mantle cell lymphoma (MCL) formation. MCL is a non-Hodgkin lymphoma of the B-cell lineage carrying the specific t(11;14) translocation between chromosomes 11 and 14, which leads to the overexpression of CCND1 [12]. MCL can be further divided into conventional (cMCL) and leukemic non-nodal (nnMCL), with 90% of patients presenting the former. We have previously identified reminiscent DNAm patterns of naive B cells (NBCs) in cMCL and memory B cells (MBCs) in nnMCL [5]. These data confirmed the common consensus that NBCs and MBCs are the respective normal counterparts of cMCL and nnMCL [13]. Moreover, DNAm analysis led to the discovery of oncogenic enhancers in MCL, exemplified by our discovery of cMCL-specific distal enhancer elements looping to the SOX11 locus in association with its expression [5, 14]. Furthermore, we have shown that the magnitude of DNAm changes can be associated with the level of proliferation experienced by MCLs, representing a powerful prognostic parameter [5, 7]. Overall, DNA methylome studies have transformed our understanding of MCL pathogenesis.

Importantly though, as for many tumors for which premalignant samples are lacking, very little is known about the epigenetic and transcriptional rewiring that occurs during early stages of MCL formation. We hypothesized, however, that DNAm patterns in full-blown tumors can aid in unraveling these. In this study, we therefore leveraged DNAm data from cMCL samples to explore its power to identify putative regulatory elements with DNA demethylation imprints of past activation. Of note, these regions are not active in full-blown tumors but display similar DNA demethylation patterns as active enhancers, indicative of their potential activation during early tumor formation. We show that they are enriched for motifs of multiple TFs, including MCL-related ones. Additionally, the putative target genes of the identified regions display an enrichment for *de novo* expression in cMCL, highlighting the potential role of these regions in early oncogene activation. Finally, we classified them into potential physiological events present in a rare subset of NBCs, which could represent the COO of cMCL, and MCL-specific pathophysiological features. Intriguingly, these two subsets of regions show distinct sets of TF motifs and target genes that, due to their identification in this study, can now be further tested for their role in early lymphomagenesis. In summary, this study focuses on DNAm from two new angles, centering around identification of putative imprints of past activation on one hand and hidden cell subtype diversity on the other hand. It not only provides new potential insights into the early steps of cMCL formation but also puts forward a new framework to study tumor formation in many more contexts. Thus, paving the way for a better understanding of the complex process of tumorigenesis.

## Materials and methods

### DNA methylation data

HumanMethylation450 BeadChip (Illumina) DNAm data of previous publications [5, 7, 15] were retrieved through the European Genome-phenome Archive (EGA). Accession numbers can be found in the Data availability section. The 450k microarray dataset comprised 475 100 CpGs from 67 samples spanning the entire B-cell differentiation lineage, including 6 hematopoietic progenitor cells (HPC), 16 early B cells, 10 naive B cells from blood (NBC-B), 5 naive B cells from tonsils (NBC-T), 9 germinal center B cells (GCBC), 10 MBCs from peripheral blood, and 11 plasma blasts and plasma cells (PB/PC) from tonsils and bone marrow, respectively, as well as 62 cMCL samples and 20 nnMCL samples (both *in silico* corrected for purity as previously described) [5]. CpG coordinates were converted to the hg38 (GRCh38) assembly using UCSC’s LiftOver tool implemented in rtracklayer (v1.62.0). The appropriate chain file was downloaded from UCSC (“https://hgdownload.soe.ucsc.edu/gbdb/hg19/liftOver/ hg19ToHg38.over.chain.gz”).

### Target CpGs selection

Mean methylation values for each CpG site across each cell population were computed. To select for CpGs that were unmethylated in cMCL and methylated in its healthy counterpart (NBC), we retained only CpGs whose mean methylation value was below 0.25 in cMCL and above 0.75 in NBC. Moreover, to correct for potential proliferation-associated drift, we selected CpGs whose methylation difference was >0.25 between each of the highly proliferative mature B-cell subtypes (GCBC, MBC, PB/PC) and cMCL.

### CpGs classification based on chromatin state annotations and chromatin accessibility

We retrieved previously published data for histone-mark-based chromatin state annotations [16, 17], comprising 2 cMCL and 3 NBC-B samples. These data resulted from the segmentation of the genome into 12 different chromatin states at 200 base pair interval, based on 6 histone marks from ChIP-seq experiments (H3K4me1, H3K4me3, H3K27ac, H3K36me3, H3K27me3, and H3K9me3), adopting ChromHMM [18]. Briefly, ChromHMM uses a multivariate hidden Markov model to segment the genome based on combinatorial patterns of histone modification signals across the genome. The resulting 12 states represent distinct combinations of histone mark enrichment, as shown in [16]. The 12 states are defined as follows: Active Promoter (E1), Weak Promoter (E3), Poised Promoter (E4), Strong Enhancer 1 (E2), Strong Enhancer 2 (E6), Weak Enhancer (E5), Transcription Transition (E7), Transcription Elongation (E9), Weak Transcription (E8), H3K9me3 Repressed (E10), H3K27me3 Repressed (E12), Heterochromatin/Low signal (E11). We applied a hierarchical classification to assign each CpG to a discrete state in cMCL and NBC-B, prioritizing active annotations over inactive ones (Supplementary Fig. S1A). The hierarchy was applied as follows: (i) Active Promoter, if at least one of the samples per population fell in the E1 category; (ii) Active Transcription, if at least one of the samples per population fell in the E7 category; (iii) Active Enhancer, if at least one of the samples per population fell in either one of the E2 or E6 categories; (iv) Other, if at least one of the samples per population fell in either one of these categories: E3, E4, and E9; (v) Primed, if at least one of the samples per population fell in the E5 category; and (vi) Inactive, if none of the samples per population fell in the categories above. Then, we simplified the categorization by merging the active categories (active promoters, active transcription, and active enhancers) into one, named active. In this project, when we refer to the chromatin state of a CpG or a set of CpGs, we refer to their chromatin state in cMCL if not otherwise specified.

To statistically assess chromatin state enrichment and depletion in the selected dataset compared to the background (all CpGs in the 450k array), a Monte Carlo simulation approach was employed. The null hypothesis assumes no differences in the annotation frequency between the selected and background datasets. To simulate the distribution of annotation counts under the null hypothesis, we conducted 100 000 independent random samplings without replacement from the background dataset, with the sample size matched to the size of the selected dataset. Then, for each annotation class, we compared its count in the sampled data with its count in the selected dataset and computed enrichment scores. To that end, the simulation was scored as 1 if the count of a given annotation class in the simulated sample was equal to or greater than that in the selected dataset; otherwise, it was scored as 0. Upon the finalization of the samplings, the scores are summed up and the *P*-values are computed as follows:


\begin{eqnarray*}
P = \frac{{r + 1}}{{n + 1}},
\end{eqnarray*}

where *r* is the number of counts and *n* is the number of permutations. The ones are added, following a common practice in permutation testing to add the actual sample as one of the permutations.

In the downstream analysis, we split the inactive and active categories further. The CpGs in inactive regions were split into two subcategories, inactive NBC-hom and inactive NBC-het, based on their clustering using the hclust function in R, which was run under default settings with the DNAm levels in the 15 NBCs as input. For each CpG in our selection, we also checked for overlaps with accessibility peaks in cMCL, which were subsequently used to split the active regions into those overlapping with accessibility peaks and the remaining inaccessible subset. Chromatin accessibility data for cMCL was retrieved from [17], coming from the same samples as the chromatin states.

### CpGs genomic location

To annotate the CpGs in relation to the genes, we used the annotatr package (v1.28.0) [19] along with TxDb.Hsapiens.UCSC.hg38.knownGene (v3.18.0). The gene annotations included intergenic, 1–5 kb upstream regions to the transcription starting sites (TSS), promoters (<1 kb upstream of the TSS), 5′ untranslated regions (5′ UTRs), first exons, exons, introns, and 3′ UTRs. If a CpG was annotated with *first exon*, the *exon* annotation was removed to avoid redundancy. Due to the possibility that multiple transcripts can be linked to one CpG, which would lead to an overrepresentation of these CpGs in the genome annotation distribution, we calculated a fractionated score for each genic annotation per CpG site, summing up to one. In this way, each CpG counts as one in the genome annotation distribution. Effectively, this score represents the ratio of the number of times a CpG site is annotated to a specific genic annotation over the total number of annotations for that CpG site. Then, for each CpG category, we summed up the fractionated genome annotation score assignments of all individual CpGs. Enrichment compared to the background (all CpGs in the 450k array) was evaluated using a Monte Carlo simulation as described for chromatin states.

### Transcription factor motif enrichment

We employed the PWMEnrich R package (v4.38.0) [20] to identify potential TF motifs associated with cMCL-specific demethylation events. We used a curated, non-redundant set of 879 PFMs (Position Frequency Matrices) from the 2024 JASPAR CORE vertebrate collection [21]. We performed this analysis separately for the CpGs belonging to the different chromatin state categories, namely the active, inactive, active accessible, active non-accessible, inactive NBC-hom, and inactive NBC-het CpG groups. The common background was composed of CpGs selected from the 450k microarray dataset, whose mean methylation values in NBCs are above 0.75. From this background, we excluded those CpGs that were already present in the selection. For both target and background sets, each CpG was extended by 50 bp on each side, using the *getSeq* function from the BSgenome package (v1.66.3) in Bioconductor. To account for 450k dataset biases, base frequencies in the background sequences were computed and used to convert the PFMs to Position Weight Matrices (PWMs). Using these PWMs and the 100-bp background sequences, we constructed a log-normal background distribution in 100-bp segments (tiles) for motif enrichment analysis. Per chromatin state category, motif enrichment was then determined within the respective target sequences by the *motifEnrichment()* function using the above-mentioned background distribution. The output provided a ranked list of TFs for each chromatin state, accompanied by *P*-values and top motif percentages, indicating the percentage of sequences in which a TF motif ranked within the top 5%. TF motifs with a top motif percentage of at least 10% and a *P*-value below .05 were selected.

### Transcription factor ChIP-seq analysis

Non-redundant ChIP-seq and ChIP-exo peak sets were downloaded from the ReMap database (all available data for human cells, identified in cell lines and primary tissues across different disease states and experimental conditions at https://remap.univ-amu.fr/storage/remap2022/hg38/MACS2/remap2022_nr_macs2_hg38_v1_0.bed.gz). For the active and inactive CpG categories, the TFs identified by the motif enrichment analysis were assessed for ReMap-based binding frequencies in the ±50 bp window surrounding the selected CpGs. Enrichment was calculated relative to the background set of CpGs used for TF motif enrichment, with *P*-values calculated using Fisher’s exact test and corrected for multiple testing using the Benjamini–Hochberg (BH) method.

### Gene expression analysis

Each CpG was assigned to the closest protein-coding gene (from the gencode release 40). Specifically, each intragenic CpG was assigned to the host gene, while intergenic CpGs were assigned to the nearest transcription start site. We downloaded RNA-seq data available from a previous study [17]. In total we retrieved data from three NBC-Bs, three NBC-Ts, three GCBCs, three MBCs, three PCs, and two cMCLs. The expected counts and fragments per kilobase of transcript per million mapped reads (FPKM) values were used in downstream analyses. The datasets were filtered to retain protein-coding genes only. For each gene and population, mean log10 (FPKM +0.01) were calculated, retrieving one gene expression value per gene per population. Genes were considered expressed in a population if their mean log10 (FPKM +0.01) was above 0 (FPKM value >1). Genes not expressed in cMCL and NBC-B were assigned to the category “not expressed” and were not considered for differential expression testing when comparing these populations. Differential expression testing comparing cMCL to NBC-B was performed on the expected count matrices using the R package DESeq2 (v1.42.1) [22], and Log_2_ FCs and adjusted *P*-values (Benjamini–Hochberg method) were calculated. Based on this analysis, genes were classified into six categories, following this hierarchy: (i) not expressed—genes not expressed in either cMCL or NBC-B; (ii) stable expression—genes with abs(log_2_ FC) <1 or adjusted *P*-value ≥.1; (iii) lost expression—genes expressed in NBC-B but not expressed in cMCL; (iv) downregulated—genes with log_2_ FC ≤−1 and adjusted *P*-value <.1; (v) *de novo* expression—genes expressed in cMCL but not expressed in NBC-B; and (vi) upregulated—genes with log_2 _FC >1 and adjusted *P*-value <.1. Genes from our selection not present in the RNAseq dataset were not considered for this analysis. We explored enrichments of these gene categories in our gene set selections (genes closest to active, inactive, active accessible, active not accessible, inactive NBC-hom and inactive NBC-het CpGs) compared to the background (the closest genes of all CpGs with mean DNAm values above 0.75 in NBCs, excluding those already present in the selection, as for the TF motif enrichment). To evaluate enrichment and depletion of the different categories compared to the background, we used a Monte Carlo simulation correcting for CpG probe density. Briefly, we conducted 10 000 independent, random CpG samplings without replacement from the background dataset, with sample size matching the size of the investigated CpG dataset. As for the investigated dataset, we then retrieved the unique set of closest genes. Finally, for each gene category, we compared its fraction in the sampled data to its fraction in the investigated dataset. Next, enrichment scores were calculated as described in the chromatin state annotation section.

### Gene Ontology and KEGG pathway analyses

Gene Ontology (GO) and Kyoto Encyclopedia of Genes and Genomes (KEGG) pathway analyses were performed using the clusterProfiler R package (v4.6.2). The background set included all *Homo sapiens* genes assigned as the closest gene to one or multiple CpGs with mean DNAm values above 0.75 in NBCs. For statistical significance, *P*-values were adjusted using the BH method. A q-value cutoff of < 0.1 was applied to each CpG subset. We implemented rrvgo (v1.14.2) [23] to group similar Gene Ontology terms. For the KEGG pathways analysis, KEGGREST (v1.42.0) was used to define categories for each term.

### Bioinformatics analyses

All analysis and visualization were performed in R (version ≥4.2). In addition to the packages mentioned above, the following R packages were used: data.table (v1.16.4), tidyverse (v2.0.0), pheatmap (v1.0.12), ggalluvial (v0.12.5), ggseqlogo (v0.2), gridExtra (v2.3), ggpubr (v0.6.0), UpSetR (v1.4.0), ComplexHeatmap (v2.18.0), RcolorBrewer (v1.1.3), GenomicRanges (v1.54.1), GenomicFeatures (v1.54.4), AnnotationHub (v3.10.1), biomaRt (v2.58.2), ggplotify (v0.1.2), patchwork (v1.3.0), BSgenome.Hsapiens.UCSC.hg38 (v1.4.5), org.Hs.eg.db (v3.16.0), and openxlsx (v4.2.8).

## Results

### Major DNA demethylation events in cMCL can occur outside active regions

**Figure 1.**
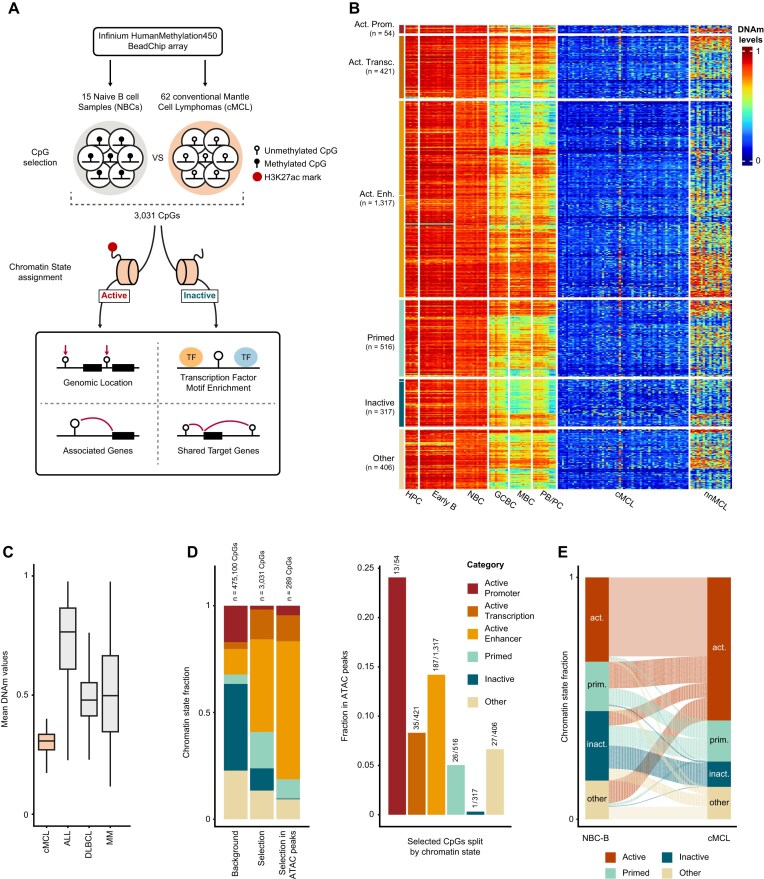
Characterization of DNA demethylation events in cMCL. (**A**) Schematic describing the workflow of the project. (**B**) Heatmap of the DNAm levels in the different healthy populations and in the two subtypes of MCL (conventional and non-nodal). The CpGs are split based on their chromatin state in cMCL. MCL data shown are corrected for tumor purity. HPC: hematopoietic progenitors cells; Early B: pre-B1 and pre-B2 cells; NBC: naive B cells from blood and tonsil; GCBC: germinal center B cells; MBC: memory B cells; PB/PC: plasmablasts/plasma cells; cMCL: conventional mantle cell lymphoma; and nnMCL: non-nodal mantle cell lymphoma. (**C**) Boxplot of the mean methylation levels of the identified CpGs in cMCL and other B-cell malignancies originating from distinct stages of B-cell differentiation. ALL: acute lymphoblastic leukemia; DLBCL: diffuse large B-cell lymphoma; MM: multiple myeloma. Of note, the data shown are not corrected for tumor purity. (**D**) Classification of the 3 031 identified demethylated CpGs (left panel, middle bar) and its subset of 289 CpGs overlapping with accessibility peaks in cMCL (left panel, right bar) based on their chromatin state in cMCL, compared to the whole set of CpG analyzed (left panel, left bar). Fraction of selected CpGs in each chromatin state category that overlaps with accessibility peaks in cMCL (right). (**E**) Alluvial plot of the 3 031 identified demethylated CpGs, showing the relationship between their associated chromatin states in naive B cells from blood (NBC-B, left) and cMCL (right). Here, active promoters, active transcription, and active enhancers are fused in a unique category called active.

To explore the DNAm landscape of cMCL in depth, specifically focusing on common DNA demethylation changes in the context of additional epigenetic layers such as histone marks and chromatin accessibility, we mined 450K DNAm data of 62 cMCL samples, collected at diagnosis, and their corresponding normal B-cell controls [5, 15] (Fig. 1A). First, CpGs showing consistent low DNAm levels in cMCL (mean <0.25) and high DNAm levels in its normal counterpart, naive B cells (NBCs, mean >0.75), were identified. Importantly, DNAm patterns of NBCs from blood (NBC-B) and tonsil (NBC-T) are highly similar and were therefore both used as controls. At the level of chromatin states and gene expression, though, they show clear differences, with cMCLs being closer to NBC-Bs [17], which were thus used as controls for those layers of information in this study. As expected, the selected CpGs did not show signs of demethylation in HPCs and early B cells (Early B) but displayed DNA demethylation in late B-cell stages associated with DNAm-based proliferation-associated drift [7]. To control for this phenomenon, the DNAm values of the selected CpGs were compared between cMCL and mature B-cell subtypes, which show a high level of this epigenetic drift, comprising germinal center and memory B cells, and plasma blasts/plasma cells (GCBCs, MBCs, and PB/PCs). In this way, we were able to subset our selection to only those CpGs with DNAm patterns specific for cMCL (*n* = 3 031, cMCL DNAm mean < 0.25, NBC DNAm mean > 0.75, DNAm difference > 0.25 between cMCL and GCBCs, MBCs, and PB/PCs) for further analyses (Fig. 1B and Supplementary Table S1). We furthermore confirmed that their DNAm loss was highly selective for cMCL in comparison to other B-cell tumors with different normal counterparts, such as acute lymphoblastic leukemia (ALL), diffuse large B-cell lymphoma (DLBCL), and multiple myeloma (MM) [7] (Fig. 1C).

Next, we aimed to understand the association between DNAm loss and genome activity and therefore classified the identified CpGs based on previously defined histone mark-based chromatin states in cMCL (Fig. 1B and D and Supplementary Fig. S1A) [16, 17]. As expected, the largest fraction of CpGs (1 792/3 031, 59.1%) resided in active genomic regions. More specifically, compared to the background chromatin-state distribution, we observed an enrichment for active enhancers (3.6-fold, from 12.0% to 43.5%, *P*-value <10^−4^) and actively transcribed regions (3.3-fold, from 4.2% to 13.9%, *P*-value < 10^−4^), as well as primed regions (3.8-fold, from 4.4% to 17.0%, *P*-value < 10^−4^) (Fig. 1D). Overall, these observations are in line with the known association between DNAm loss and genome activation, especially at enhancer regions [4]. Next, we assessed whether the selected CpGs tend to cluster together in the genome. As expected, a high fraction of CpGs within the active promoter category (22.2%) are located within 50 base pairs of each other, while this percentage drops to less than 10% for CpGs in other categories (Supplementary Fig. S1B), suggesting that these DNA demethylation events are largely independent.

The detected subset of 317 demethylated CpGs located in inactive genomic regions in cMCL specifically caught our attention, deviating from the expected pattern that DNA demethylation is associated with genome activation. To explore these CpGs further and to determine whether their loss of DNAm could be explained by the sole presence of chromatin accessibility without other activation marks, we turned to chromatin accessibility data [17]. Overall, a small number of all identified demethylated sites (*n* = 289/3 031, 9.5%) resided within cMCL-associated chromatin accessibility peaks. However, as expected, the majority of these (235/289, 81.3%) overlapped with active regions. In fact, except for one CpG, the inactive CpGs did not show any association with chromatin accessibility in cMCL, which therefore cannot explain their loss of DNAm (Fig. 1D). Furthermore, most of the inactive regions (287/317, 90.5%) were also inactive in NBC-B (Fig. 1E), which is expected based on their high DNAm values (>0.75) in this population. Overall highlighting that, at the bulk level, no evidence exists that these regions represent active chromatin in cMCL or its normal counterpart. Finally, we investigated the genomic location of the CpGs from our selection that fall within inactive regions, comparing them to the distribution of all CpGs in the background set. We observed an enrichment for intergenic regions (1.44-fold, from 9 to 13%, *P*-value = .04), introns (1.26-fold, from 39 to 49%, *P*-value <10^−4^), and distal promoter regions (1–5 kb upstream of promoters, 1.5-fold, from 14% to 21%, *P*-value <10^−4^) (Supplementary Fig. S1C). All these locations can harbor distal regulatory elements with the potential to enhance gene expression. In summary, while DNA demethylation in cMCL is highly associated with genomic activation of enhancer regions, a small set of CpGs displaying similar patterns of DNAm loss reside in inactive, inaccessible regions with unknown but potentially relevant functions in the context of cMCL pathogenesis.

### DNAm loss at inactive loci could represent histories of gene regulatory events

**Figure 2.**
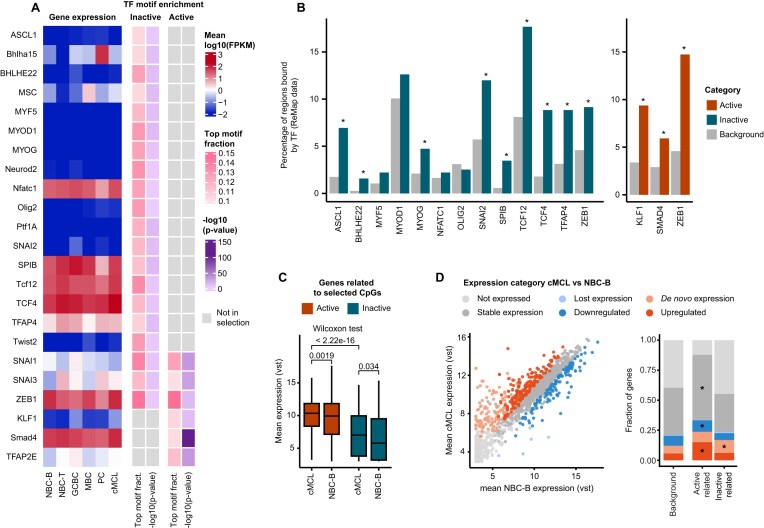
DNAm loss in cMCL in relation to transcription factor motifs and cMCL-associated gene expression events. (**A**) Transcription factors whose motifs are enriched around the identified CpGs (± 50 bp) in the active and inactive CpG selection. Gene expression values, as well as −log10 (*P*-values), and the proportion of sequences in which the motif was among the top 5% are shown. For Ptf1A, multiple motifs were enriched, but only the highest-ranked motif is listed. Please refer to Supplementary Fig. S2A and Supplementary Table S2 for all motifs. NBC-B: naive B cells from blood; NBC-T: naive B cells from tonsil; GCBC: germinal center B cells; MBC: memory B cells; PC: plasma cells; and cMCL: conventional mantle cell lymphoma. (**B**) Percentage of regions bound by the detected TFs based on ReMap data. Information on SNAI3, BHLHA15, MSC, NEUROD2, PTF1A, TWIST2, and TFAP2E was not available in the ReMap dataset. SNAI1 was only associated with 4 CpGs in the background set and therefore excluded as well. (**C**) Boxplot of the mean gene expression levels for the genes assigned to the categories of active and inactive CpGs. (**D**) Scatterplot of the mean gene expression levels in cMCL and NBC-B. Each gene is labeled based on its expression category in cMCL compared to NBC-B (left). Expression category distribution for the genes in our selection compared to the background. Asterisks highlight enrichment (*P*-value <.05), calculated using a permutation test (right).

We next aimed to investigate how the inactive subset of regions that consistently lose DNAm in cMCL could functionally relate to cMCL formation. To explore this, we performed TF motif enrichment analysis, separating the CpGs falling within active and inactive regions. By employing PWMEnrich [20], we detected 25 enriched TF motifs that passed our selection threshold (present in the top 5% of motifs in at least 10% of the sequences and a *P*-value <.05). The TFs associated with the identified motifs did not show a cMCL-exclusive expression pattern but tended to be either expressed in all mature healthy B-cell differentiation stages and cMCL or in none of them (Fig. 2A and Supplementary Table S2). This indicates that overall, the observed enrichment of TF motifs in our selected regions cannot be explained by cMCL-specific TF expression. Of note, many of the detected transcription factor motifs share the E-box sequence (CANNTG) (Supplementary Fig. S2A). The group of active CpGs showed enrichment for the motif of the MCL-associated E-box TF ZEB1, in line with previous findings [7, 24]. Surprisingly though, the enrichment for this MCL-associated TF motif was not limited to the active CpG group but could also be observed for the inactive CpGs. In addition, in the inactive set of CpGs we detected enrichment for the MCL-related E-Box TF TCF4 [7]. These findings strengthen our hypothesis that these regions, although inactive in the full-blown tumor, may have undergone TF-mediated DNA demethylation in the past, pointing towards a potential functional role in cMCL pathogenesis. To further support our findings, we downloaded ChIP-seq and ChIP-exo data from the ReMap database, a highly valuable resource of TF binding sites derived from over 8 000 chromatin immunoprecipitation experiments in cell lines and primary tissues across different disease states and experimental conditions [25]. Using these data, we observed a significant binding enrichment for many identified TFs compared to the background (Fig. 2B). This overlap with experimental data strengthens our hypothesis that our selected regions may be bound by the identified TFs along the trajectory of lymphomagenesis. Intrigued by the identification of many DNA demethylation events in active regions outside chromatin accessibility peaks (Fig. 1D), we additionally took the opportunity to refine our TF motif enrichment analysis. We observed that the active inaccessible regions showed similar motif enrichments to the full set of active CpGs. In contrast, the active accessible regions displayed enrichment for motifs of many more TFs, including those associated with cMCL pathogenesis, such as TCF4 and the NF-kB-associated TF RELA [7, 26]; B-cell receptor signaling mediators such as the EGR family [27]; and KLF transcription factors with roles in the immune system and cancer [28] (Supplementary Fig. S2B and Supplementary Table S2). Overall suggesting that DNA demethylation events in regions marked by H3K27ac and chromatin accessibility in cMCL represent regulatory elements playing a prominent role at the full-blown tumor stage.

To further characterize the potential function of our selected regions in cMCL pathogenesis, we mapped them to their closest protein-coding gene. To that end, each intragenic CpG was assigned to its host gene, while intergenic CpGs were assigned to the nearest transcription start site. From now on, we will refer to the identified genes as active-related and inactive-related genes, based on the category of their associated CpGs. As expected, the active-related genes showed overexpression in cMCL compared to NBC-B. Interestingly, this pattern was also observed for the inactive category (Fig. 2C). To further understand this phenomenon, we next assigned each gene to a specific category, based on expression in NBC-B and cMCL (not expressed, stable expression, *de novo* expression/upregulated in cMCL, and lost expression/downregulated in cMCL) (Fig. 2D and Supplementary Table S3). Using these assignments, we analyzed the enrichment in our gene selections compared to the background (all the genes close to CpGs in our background set and present in the RNA-seq data) (Fig. 2D). The active-related genes were clearly enriched for expression upregulation in cMCL (fold change 2.7, *P*-value <10^−4^), which was even more pronounced for the genes associated with active accessible regions (fold change 3.5, *P*-value <10^−4^). The latter furthermore displayed an enrichment for *de novo* expression in cMCL (fold change 2.0, *P*-value <.002) (Supplementary fig. S2C). Overall, this confirms that DNA demethylation in active regions can be associated with cMCL-related gene expression. More intriguing, the inactive-related genes also showed a significant enrichment for *de novo* expression in cMCL (fold change 1.7, *P*-value 0.021). We speculate that this enrichment could represent the establishment of tumor-specific gene expression programs prior to full tumor formation.

### DNAm loss hints at putative TF and gene expression programs along lymphomagenesis

**Figure 3.**
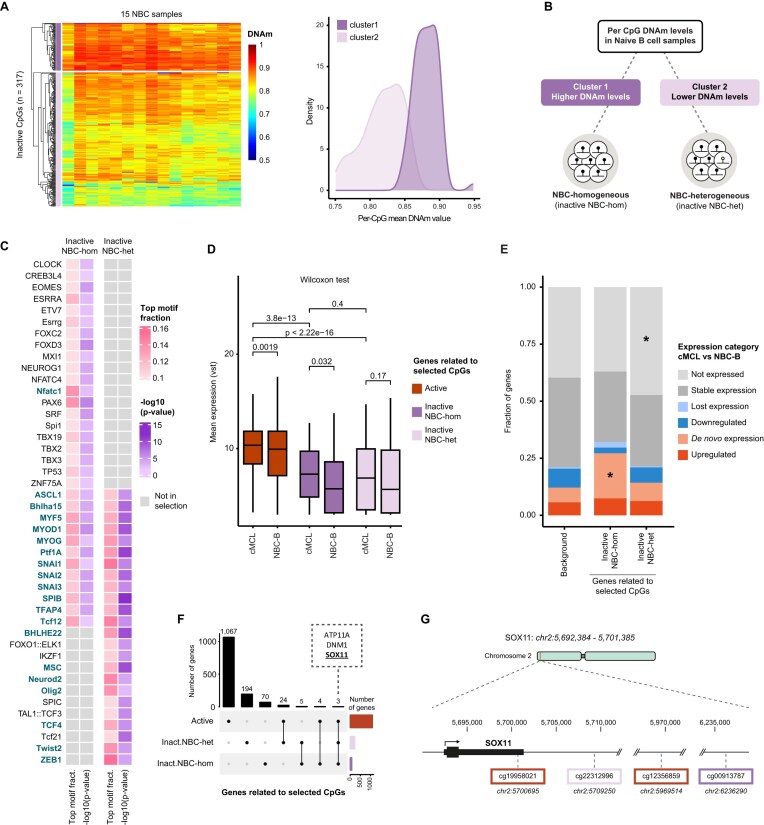
Demethylation at inactive sites could represent genomic activation along lymphomagenesis. (**A**) Heatmap of DNAm levels for the 317 inactive CpGs across 15 NBC samples. Hierarchical clustering identifies two main groups (left). Distribution of per-CpG mean NBC DNAm values within each cluster (right). (**B**) Classification workflow for inactive CpGs based on the clustering across NBC samples. (**C**) Transcription factors whose motifs are enriched around the identified CpGs (± 50 bp) in the inactive NBC-hom and inactive NBC-het CpG selection. For Ptf1A, multiple motifs were enriched, but only the name of the highest-ranked motif is listed. Please refer to Supplementary Table S2 for all motifs. (**D**) Boxplot of mean gene expression levels for genes assigned to the categories of active, inactive NBC-hom, and inactive NBC-het in both NBC-B and cMCL. (**E**) Expression category distribution for the genes in our selections compared to the background. (**F**) Upset plot of the identified genes split per category (active, inactive NBC-hom, and inactive NBC-het) ordered per numerical representation. Most genes are specific for a single category, with a minor degree of overlap. Highlighted in the box are the ones that are found in all three categories. (**G**) Graphical representation of SOX11 and its assigned CpGs (hg38). The CpGs are primarily located in the intergenic region downstream of the gene, except for one located in its 3′UTR.

The enrichment of TF motifs related to cMCL and the immune system in consistently demethylated, inactive genomic regions in cMCL, together with the association to cMCL-specific gene expression patterns, suggests that these regions may play a gene regulatory role that is relevant for cMCL pathogenesis. We speculate that their DNA demethylation could be a reminiscent feature of genomic activation prior to full-blown tumor formation. Importantly, while their DNA demethylation could represent cMCL-specific epigenetic events occurring during early tumor stages, it could also reflect the epigenetic state of the COO that gives rise to cMCL. Currently, the COO of cMCL is considered to be the global NBC population [13]. Bulk DNAm values in NBCs, however, show certain, subtle deviations from the expected DNAm level distribution, potentially representing the presence of so far uncharacterized cell subtypes within this population [15]. More specifically, the presence of small fractions of cell subtypes with specific demethylation patterns will slightly lower bulk DNAm levels, which overall tend to be high in NBCs. These moderately lower DNAm levels may thus reflect hidden heterogeneity within the NBC population with potential relevance for the origin of tumors. To explore the possibility that our 317 inactive CpGs could represent such NBC-related cell subtype diversity, we further checked their DNAm patterns in NBCs. Unsupervised clustering divided them into two main clusters, one with higher (cluster 1) and a second one with lower (cluster 2) DNAm values in NBCs (Fig. 3A). Next, we used this clustering to split our inactive CpGs into two subgroups (Fig. 3B). CpGs with lower DNAm values in NBC were classified as NBC-heterogenous (NBC-het, n = 234), referring to the fact that they may represent heterogeneity in NBC cell subtype populations as explained above, while those with higher DNAm values were classified as NBC-homogenous (NBC-hom, n = 83). We also calculated the DNAm value that best splits the CpGs, which we observed to be at 0.86 (data not shown). Notably, this value was not used to split the CpGs but only determined to give a global notion of the DNAm values within the two groups. The NBC-het group possibly represents a DNA demethylation signature present in a small NBC subpopulation that acts as cMCL’s COO, while the NBC-hom subset might capture DNA demethylation events that are absent in NBCs but specifically occur early during cMCL development.

To understand which TFs could be active in the COO of cMCL or during early lymphomagenesis, we repeated the TF motif enrichment analysis. We discovered that TCF4 and ZEB1 motif enrichment specifically occurred in the inactive NBC-het CpGs (Fig. 3C and Supplementary Table S2). Based on this finding, we speculate that these TFs are expressed and bind to genomic regions with non-oncogenic functions in the COO of cMCL, while, during late stages of disease development, they might adopt a tumorigenic role by activating cMCL-specific regulatory elements. NBC-het specific patterns were also observed for various B-cell differentiation-related TFs, including IKZF1 and MSC. In addition, the association of this CpG category with FOXO1 is of interest, since it is particularly expressed in B1 cells [29]. As B1 cells represent a subset of NBCs that has been proposed as a possible COO of cMCL in murine models [30], our findings may potentially relate to events playing a role in this cell subpopulation linked to cMCL. Another category, the immune-cell-related TFs SPIB and TFAP4, were enriched in both the NBC-het and NBC-hom categories, suggesting that they may play a dual role, affecting both the COO and early disease stages of cMCL. Among the NBC-hom-related TFs, we also observed EOMES, which is specifically expressed in cMCL (data not shown) and has been linked to immunosuppressive activity enhancing IL-10 expression [31]. Moreover, in this category we found motifs of NFAT and TBX family members to be enriched. These two families of TFs have been shown to be aberrantly activated in different lymphoid malignancies [32, 33].

We also refined our gene expression analysis by splitting the assigned genes into inactive NBC-het-related and NBC-hom-related gene categories. In this way, we observed that the inactive NBC-hom-related genes showed a significant overexpression in cMCL (*P*-value .032, Fig. 3D) and an enrichment for *de novo* expressed genes (fold change 3.1, *P*-value <10^−4^, Fig. 3E). In contrast, the inactive NBC-het group did not display links to cMCL-related events. This finding suggests that inactive NBC-het DNA demethylation events, which we consider to be potentially linked to the COO of cMCL, overall do not relate to gene expression events in cMCL. Next, we looked further into our putative target genes using gene ontologies and KEGG pathway analyses (Supplementary Table S4 and Supplementary Fig. S3). As expected, the active-related genes were linked to cMCL-associated features such as B-cell receptor signaling, B-cell activation, and NF-kB signaling [26]. Moreover, we detected terms related to Notch signaling, which could link to cMCL, as Notch genes are known to be commonly mutated in this tumor [12, 34]. Focusing on the inactive related genes, only the NBC-het categories showed enrichment of few pathways, either related to specific signaling events or longevity regulation. Overall, showing that while active-related genes are clearly linked to cMCL features, genes related to inactive categories tend not to show cMCL-specific characteristics based on pathway enrichment analyses.

While our analysis identified multiple putative target genes, interpreting the genes individually remains challenging. Nonetheless, we want to highlight a couple of examples to illustrate the potential biological relevance of these regions. For instance, the presence of three out of the five genes belonging to the EPHB family (EPHB2/3/6) among the inactive NBC-het-related genes is of interest. This family is related to B- and T-cell activation and regulation [35]. Moreover, we observe demethylation of CpGs from the inactive NBC-het subgroup close to MYEOV. This finding is relevant from the perspective of translocation formation, which occurs more frequently in active regions [36]. It may suggest that the MYEOV locus is active in the COO of cMCL, favoring translocation t(11;14) formation that occurs just downstream of MYEOV. Among the inactive NBC-hom-related genes, we want to highlight the presence of proteins involved in interactions with the microenvironment, such as ADAM12, ITGA7, SH3PXD2A, and TNXB, since the tumor microenvironment is known to play a key role in MCL growth and therapy resistance [37, 38]. Finally, we observed that some genes were assigned as targets of multiple DNAm sites belonging to different categories (Fig. 3F). Strikingly, one of these genes is SOX11, a well-known oncogene in cMCL (Fig. 3G) [13]. Based on our findings, we speculate that it may be regulated by different genomic regions during various stages of disease development, i.e. in its COO, during early tumor formation, and in full-blown tumors. Such transient dynamicity of gene regulatory element usage has been proven to play key roles in differentiation models, fine-tuning gene expression during cell-state transitions [39]. In summary, in this study we observed that inactive demethylated regions in cMCL may harbor relevant tumor-related information, both in terms of TF motifs and possible target genes. These DNA demethylation features, often considered passive bystanders in tumor formation, might instead be related to the disease. We provide evidence that this information can be used to infer putative genomic activation histories, providing a new approach to build novel, focused hypotheses that can be explored in follow-up studies to understand the actual epigenetic and transcriptomic rewiring along tumorigenesis.

## Discussion

DNAm patterns have been studied in the context of cancer from many different perspectives, covering early studies on tumor suppressor gene silencing [6] to more recent studies focusing on patterns associated with tumor-specific genome activation and proliferation-associated epigenetic drift [1, 5, 7]. In this study, we have explored the DNAm layer from two new angles that, to the best of our knowledge, have not been explicitly addressed before in the context of tumorigenesis. More specifically, we first focused on the study of demethylation at inactive genomic regions, including the identification of TF motifs and putative target genes. Second, we placed these findings in light of the potential existence of rare healthy cell subtypes, allowing us to build hypotheses regarding their role in the COO of cMCL or during early tumor formation.

The study of DNAm patterns in inactive genomic regions has been explored in non-tumoral contexts such as development, allowing for the identification of DNAm imprints related to germ layer-specific enhancer activity during preceding cell stages [9]. In analogy to this, we provide evidence that inactive demethylated sites in tumors may represent regions that were active in their COO or during early tumor formation. Particularly, the identification of TF motif enrichment highlights that these are not random genomic regions, but that they may represent regulatory elements bound by TFs during particular stages of tumorigenesis. Interestingly, these comprise known MCL-related TFs such as TCF4 and ZEB1 that, beyond their function in cMCL, may thus also play a role in its COO. Furthermore, we have defined additional TFs that may play a role in cMCL lymphomagenesis, such as SPIB and FOXO1. While little is known about their role in MCL, they relate to vulnerabilities that can be exploited in the context of treatment. More specifically, upregulation of SPIB has been associated with cytarabine resistance in MCL [40] and FOXO1 inhibition with suppression of MCL progression [41].

**Figure 4.**
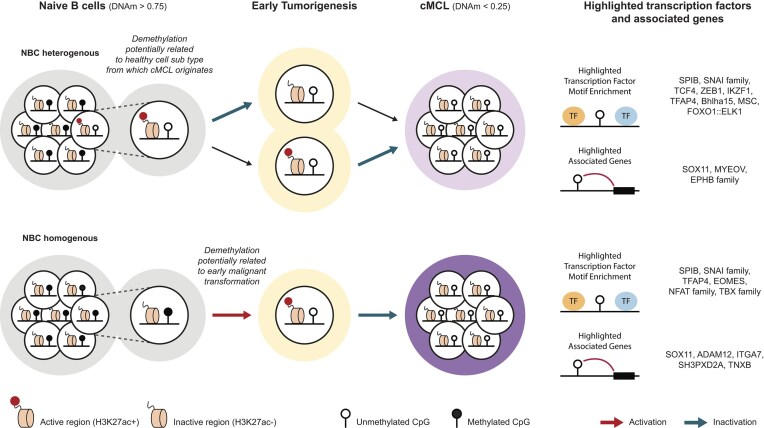
Model regarding the inactive demethylated CpGs in cMCL. We propose a possible model for cMCL-specific demethylation within inactive regions in cMCL related to its healthy COO or early stages of formation. We hypothesize that inactive NBC-heterogeneous CpGs have slightly lower DNAm levels in NBC-B, potentially representing cell subtype diversity in the bulk population related to the COO of cMCL (left, upper part), while the MCL-related CpGs have higher DNAm levels (left, lower part). While COO-related CpGs face inactivation during early or late tumorigenesis (middle panel, upper part), MCL-related ones undergo a first round of activation during early tumorigenesis, followed by later inactivation (middle panel, lower part). In the right part of the figure, the main putative transcription factors and genes of interest related to the inactive CpGs are highlighted.

The biological relevance of our approach can be appreciated from its power to generate new, explorable hypotheses in the context of tumorigenesis, for instance in light of the COO. While the COO concept is well known in the field of tumor formation, COO classifications for many neoplasms remain currently limited to broad cell types, such as NBCs for cMCL and MBCs for nnMCL. Large-scale single-cell initiatives, such as the Human Cell Atlas [42], however, show that many more cell subtypes may exist that have not been characterized yet. It is not unlikely that these represent the COO of specific tumor entities. In this study we show that major demethylation events at a significant amount of CpGs in cMCL samples could represent an imprint of the DNAm signature in its COO, linked to specific TFs and downstream target genes. This knowledge largely facilitates a focused search for these rare healthy subtypes in healthy donors, using high-sensitive targeted single-cell methods, including scDNAm technologies [43, 44]. An additional important implication of our work hints to the fact that we likely need to work on a new consensus regarding epigenetic and transcriptomic rewiring observed in tumor samples. More specifically, our study suggests that these events need to be subclassified as physiological in the case of COO signals and pathophysiological in the case of tumor-specific events based on high-resolution single-cell and experimental follow-up studies, overall providing a new and better understanding of tumor formation (Fig. 4).

Finally, we would like to speculate what the activation of genomic regions during early tumor formation could mean at the individual target gene level, especially when these regions are inactive at later stages. First, they may regulate the expression of genes that are only essential during early tumor formation. The expression of the affected genes may be dispensable or even give rise to selective disadvantages at later tumor stages. It will be difficult to trace those transcriptomic hits, as they cannot be observed in full-blown tumor samples, but pre-malignant model system generation offers opportunities to test their role in early tumor formation. Second, the identified putative genome activation events could aid the activation of essential proto-oncogenes during early tumor stages, while at later stages secondary regulatory mechanisms may take over to maintain proto-oncogene expression. For instance, in the case of SOX11, such secondary regulatory events could be established through positive feedback loops via SOX11 binding to its own MCL-specific enhancer [5, 14]. This scenario of temporal usage of multiple different enhancers to regulate gene expression is clearly established in the context of development [8, 39, 45, 46]. It is thus well possible that similar processes exist for proto-oncogene activation and expression maintenance during tumorigenesis. In this regard, our approach helps to identify the potential regulatory regions to be tested in follow-up experiments. In summary, our results provide important new insights into cMCL formation and the putative TFs playing a role during early stages of its formation, as early as in its COO. Beyond this, we put forward a framework that provides opportunities to identify histories of putative regulatory element activation in many other tumors for which DNAm and chromatin state data are available. Thus, overall, allowing the generation of new, promising hypotheses for follow-up studies to aid early detection, pre-malignant model generation, and better understanding of tumor formation.

## Limitations

The outcomes of our analyses allow us to build plausible hypotheses in the context of cMCL lymphomagenesis; however, further functional studies are needed for their validation. Follow-up experiments extend to different areas as explained next. First, targeted single-cell studies are needed to identify B-cell subpopulations with MCL-like features in healthy individuals. Their identification and full molecular characterization then need to be followed by isolation, manipulation—e.g. introducing the translocation t(11;14)—and transplantation studies in mouse models to investigate their role in MCL pathogenesis. Second, dCas9-based genome activation experiments in *in vitro* B-cell differentiation models will aid to identify the role of the putative enhancers in target gene activation. Importantly, these studies need to include the analysis of the 3D genome layer. This was omitted in the current manuscript, which is a clear limitation in the context of linking DNA demethylation events to their actual target genes, although many identified CpGs are located within genes, which often represent their targets.

At the level of individual regions, the distinction of demethylation due to enhancer activation from that caused by epigenetic drift is challenging. Therefore, we cannot fully exclude that epigenetic drift plays a role in a fraction of DNA demethylation events, especially in inactive regions. We have accounted for this in multiple ways, though. First, we focused on demethylation events that occur consistently in 62 cMCL samples, which are highly unlikely to be caused by rather random processes such as epigenetic drift. Second, we exclude CpGs that show low DNAm levels in B-cell populations suffering from high epigenetic drift. Nevertheless, we have adopted a rather relaxed threshold to correct for this, as it is known that epigenetic drift can affect regions, such as the SOX11 enhancer, that are inactive in normal B-cell subtypes but active in cMCL. An additional limitation of our study is that the presence of TF motifs does not represent actual TF binding. The ReMAP TF binding database, however, confirmed binding for most of our TFs, though not necessarily in MCL cells. A further complication is that multiple TFs can have similar binding motifs. Moreover, dependent on the tools, background, and cutoff used for detection, TF motif enrichment results may slightly vary. Hence, while global TF patterns have been elucidated in our study, the actual events related to DNA demethylation at individual loci need to be further explored.

Finally, the applicability and validation of our approach is dependent on the availability of uniformly generated DNAm data from many patients and healthy cells of matching tissues, as well as the presence of chromatin state data. Hence, overall our method may not be applicable to many tumors at present. In line with this, the lack of external large-scale DNAm datasets for cMCL limits the possibility to test the validity of our results in independent cohorts. However, as more data are generated every day, we believe our approach could become useful for many tumors in the near future. Additionally, it is important to highlight that for each new dataset the clustering-based identification of CpGs that may represent hidden cell subtype heterogeneity, i.e. those showing a slight decrease in DNAm compared to the overall high DNAm values in other CpGs, may lead to similar but not the exact same thresholds in DNAm values, which will depend on the CpGs included and the type of data analyzed. Of further note, our study has focused on the set of CpGs covered by the 450k methylation array, lacking the ability to map the presence of DNA demethylation signals in uncovered regions. This is particularly important when one wants to study individual gene activation dynamics, because part of the regulatory elements are likely missing. To provide a full picture of the DNAm landscape, genome-wide detection methods such as WGBS are needed. The small number of patient samples screened by such methods, however, remains a limiting step so far, as DNA demethylation consistency across patients cannot be assured. Nevertheless, despite all the limitations listed above, we believe that our cMCL-specific results as well as the general framework we put forward will aid the study of tumorigenesis.

## Supplementary Material

lqaf187_Supplemental_Files

## Data Availability

All data used in this study were mined from previous studies and can be found under the following accession numbers: EGAS00001001196, EGAS00001001637, EGAS00001001596, EGAS00001004640, EGAS00001000327, and EGAS00001000841. The code and a test dataset are available at https://doi.org/10.6084/m9.figshare.30178684.
