# Supplementary material for: DNA methylation profiles aid to identify putative genome activation histories along lymphomagenesis
Source: NAR Genom Bioinform. 2026 Jan 10;8(1):lqaf187. doi: 10.1093/nargab/lqaf187 (PMC12789802; doi:10.1093/nargab/lqaf187)
Supplement: lqaf187_Supplemental_Files [file lqaf187_supplemental_files.zip › SupplementaryData_NARGAB_20251007.pdf]

Supplementary data belonging to manuscript by Albinati *et al*, entitled:

DNA methylation profiles aid to identify putative genome activation histories along lymphomagenesis

Contents

Supplementary Table Legends........Page2

Supplementary Figure 1........Page3

Supplementary Figure 2........Page4

Supplementary Figure 3........Page5

Supplementary Table Legends

Supplementary Table 1. CpG selection overview. For each CpG in our selection, we provide the name and coordinate, the mean DNAm values for the different healthy B-cell populations and cMCL, the chromatin state category in both NBC-B and cMCL, the overlapping protein-coding gene (if intragenic) or the closest protein-coding based on TSS location (if intergenic), the overlap with ATAC peaks in cMCL, the category based on DNAm behaviour in NBC (inactive NBC-hom and –het), and the score for the different genomic locations. HPC – hematopoietic progenitor cells; S2 - pre-B1 cells; S3 - pre-B2 cells; S4 – immature B cells; NBC – naive B cells from blood and tonsil; NBCB – naive B cells from blood; NBCT – naive B cells from tonsil; GCBC – germinal center B cells; MBC - memory B cells; PB_PC – plasmablasts and plasma cells; cMCL – conventional mantle cell lymphoma.

Supplementary Table 2. Transcription factor motif enrichment. Output of the transcription factor (TF) motif enrichment analysis performed with PWMEnrich for the different CpG categories (active, inactive, active accessible, active not accessible, inactive NBC-hom, inactive NBC-het). For each TF we represent the p-value and the proportion (prop) of sequences for which the TF is in the top 5% of motifs detected.

Supplementary Table 3. Overview of putative target genes. List of all active-accessible-related, active-not-accessible-related, and inactive NBC-hom-related and NBC-het-related genes. For each gene, we provide the Ensembl gene ID and name, their chromatin-state based category, the expression levels in NBCB and cMCL, the log2FC between cMCL and NBC-B and the related adjusted p-values, and the expression category. Genes linked to multiple CpGs belonging to different chromatin-state based categories appear multiple times. NBCB – naive B cells from blood; cMCL – conventional mantle cell lymphoma.

Supplementary Table 4. Gene ontology terms and KEGG pathways. All terms enriched in the biological processes-based gene ontology analysis and the KEGG pathway analysis are shown for the three sets of genes belonging to the three analysed CpG categories (active, inactive NBC-hom, inactive NBC-het). If empty, no significant results were obtained.

Supplementary Figures

Supplementary Figure 1

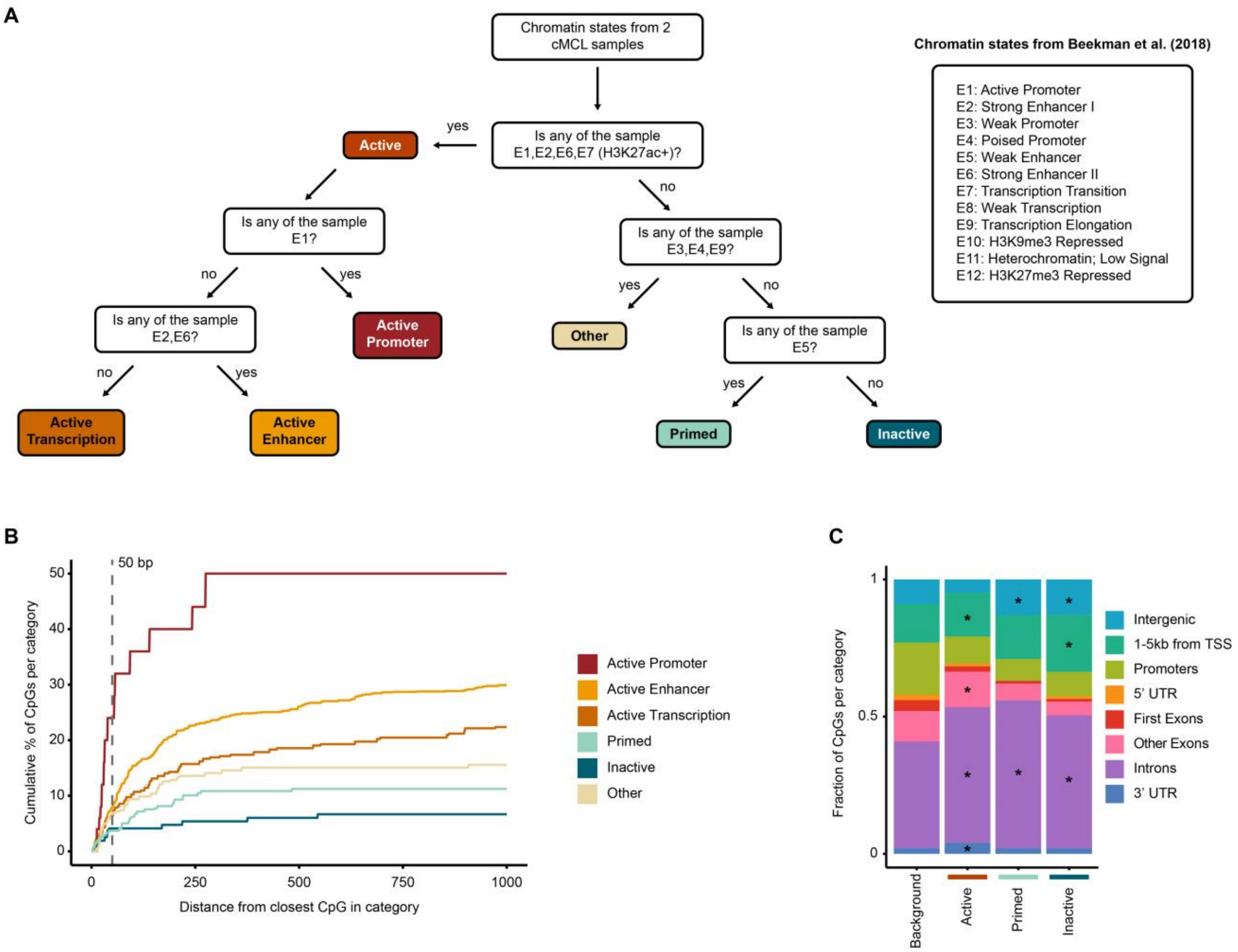

Supplementary Figure 1. CpGs selection and characteristics. (A) Decision tree used to assign each CpG to one of six categories based on chromatin states from two cMCL samples. (**B)** Cumulative percentage of CpGs located within increasing distances (in bp) of the nearest CpG in the same category. (**C)** Genomic distribution of CpGs in each category and the background. The barplot shows the fraction of CpGs in different genomic regions. Asterisks highlight enrichment with p-value < 0.05, calculated using permutation test.

Supplementary Figure 2

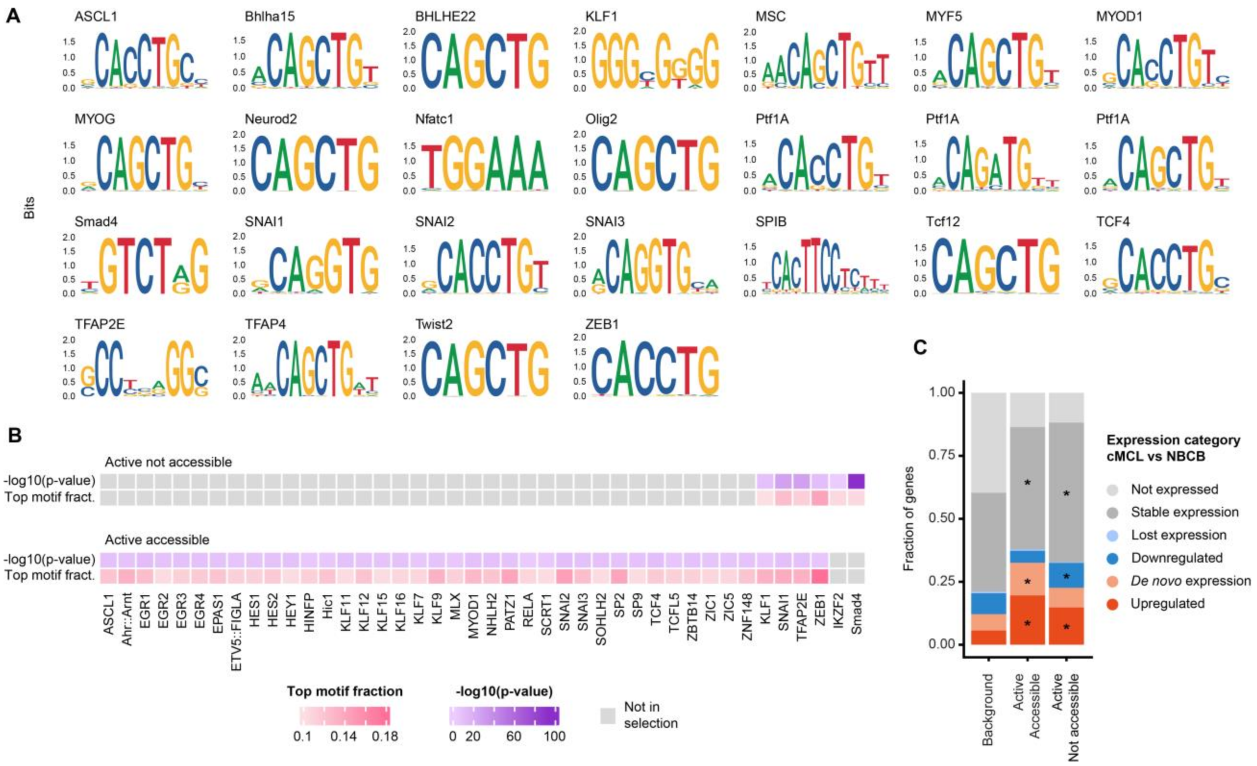

Supplementary Figure 2. TF motifs and results of active CpGs based on chromatin accessibility (A) Transcription factor (TF) motifs for the TF enriched in active and inactive CpGs. **(B)** TF motif enriched surrounding active accessible and active inaccessible CpGs. **(C)** Expression category distribution for the genes associated to active accessible and active not accessible CpGs compared to the background. Asterisks highlight enrichment with p-value < 0.05, calculated using permutation test.

Supplementary Figure 3

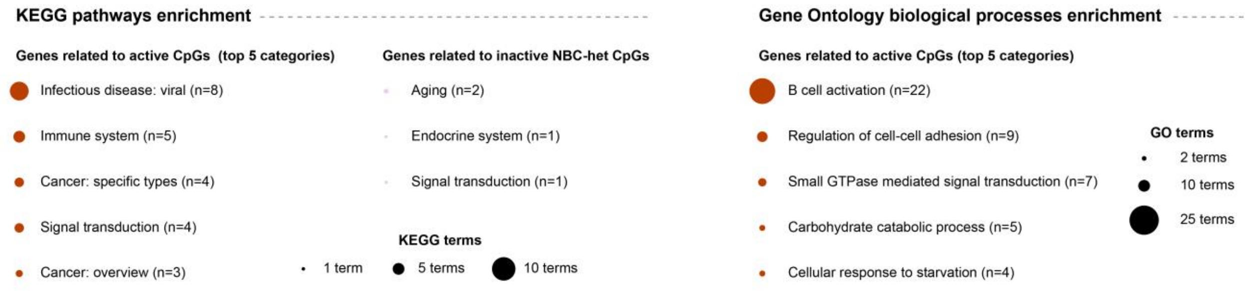

Supplementary Figure 3. Gene ontology and KEGG pathway analysis results. Summary of gene ontology (GO) enrichment for biological processes, as well as KEGG pathways. The top 5 categories are shown for active-related genes. We refer to Supplementary Table 4 for complete results. The size of the dots represents the number of terms associated with the category.
